# Patched Targeting Peptides for Imaging and Treatment of Hedgehog Positive Breast Tumors

**DOI:** 10.1155/2014/525680

**Published:** 2014-09-08

**Authors:** Daniel Smith, Fanlin Kong, David Yang, Richard Larson, Jennifer Sims-Mourtada, Wendy A. Woodward

**Affiliations:** ^1^Department of Radiation Oncology, The University of Texas M.D. Anderson Cancer Center, Houston, TX 77030, USA; ^2^Department of Cancer Systems Imaging, The University of Texas M.D. Anderson Cancer Center, Houston, TX 77030, USA; ^3^Center for Translational Cancer Research, Helen F. Graham Cancer Center and Research Institute, Christiana Care Health System, 4701 Ogletown-Stanton Road, Newark, DE 19713, USA

## Abstract

High tumor hedgehog expression is correlated with poor prognosis in invasive ductal carcinoma. Peptides which bind the patched receptor have recently been reported to have a growth inhibitory effect in tumors with activated hedgehog signaling. We sought to examine growth inhibition with these peptides in breast cancer cells and use these peptides as molecular imaging probes to follow changes in hedgehog expression after chemotherapy. Significant growth inhibition was observed in breast cancer cell lines treated with PTCH-blocking peptides. Significant* in vitro* uptake was observed with both FITC- and ^99m^Tc-EC-peptide conjugates.* In vivo* imaging studies displayed greater accumulation of ^99m^Tc-labeled peptides within tumors as compared to adjacent muscle tissue. Patched receptor expression increased after treatment and this correlated with an increase in tumor radiotracer uptake. These studies suggest that peptides which bind the sonic hedgehog docking site in patched receptor correlate with patched expression and can be used to image patched* in vivo*. Further, our data suggest that radiolabeled peptides may enable us to examine the activity of the hedgehog signaling pathway and to evaluate response to anti-cancer therapies.

## 1. Introduction

The hedgehog (Hh) signaling pathway plays a critical role in embryonic development and wound healing, and its aberrant activity is associated with several malignancies. Recent studies implicate Hh signaling in breast cancer growth and metastasis, and high tumor sonic hedgehog (SHh) expression is correlated with poor prognosis in invasive ductal carcinoma. SHh binds to the suppressive receptor patched-1 (PTCH-1) and relieves the inhibition of the transmembrane protein smoothened (Smo) by PTCH-1, resulting in the translocation of Gli transcription factors to the nucleus and activation of Hh target genes. In tumors with activated Hh signaling, high levels of PTCH-1 have been reported, especially within the tumor stroma.

Previously, we demonstrated strong detection of tumor xenografts using an iodinated derivative of the PTCH-1 binding ligand, sonic hedgehog [1]. Although this agent was capable of delineating tumor tissue, its clinical utility is limited due to poor stability and pharmacokinetics. Imaging with radiolabeled peptides has been shown to improve pharmacokinetics and the targeting of other tumor-based receptors. Therefore, we sought to develop radiolabeled peptides which dock inside the PTCH receptor. Nakamura et al. previously reported the synthesis of several peptides targeting the PTCH-1 receptor [2]. These peptides were shown to bind to the PTCH-1 receptor on the surface of pancreatic tumors and decrease tumor growth.

Here, we selected technetium-99 m (^99m^Tc) as the radioisotope because of its favorable physical characteristics for diagnostic imaging studies and due to the ease of using its benchtop generator-based system for clinical applications. It emits 140 keV gamma ray, with an 89% branching fraction, which can be detected by single photon emission computed tomography (SPECT). In addition, the half-life of ^99m^Tc is relatively long (6.02 h) compared to most nuclear imaging radioisotopes, which facilitates serial imaging that may improve the differentiation of tumor from inflammation. To label the peptide with ^99m^Tc, the chelator N,N′-ethylene-di-*L*-cysteine (EC) is selected and used as a linker. EC is known to chelate ^99m^Tc stably owing to the efficient binding of the oxotechnetium group to the two thiols and two amine nitrogen atoms of EC.

Here, we report the radiolabeling of these peptides to detect the PTCH receptor on breast cancer cells and breast cancer stem cell-enriched populations. These molecular imaging probes have the potential to identify Hh-induced changes in PTCH-1 expression, which is useful for the imaging of aberrant Hh signaling in malignancies.

## 2. Methods

### 2.1. Peptides

PTCH-binding peptides A—sequence FAPVLDGAVSTLLGV—and B—sequence DNTRYSPPPPYSSHS—were commercially synthesized with or without an N-terminal FITC-Ahx modification (GenScript, Piscataway, NJ). Peptides were resuspended at a stock concentration of 200 *μ*M in 10% DMSO in deionized water.

### 2.2. Synthesis and Radiolabeling of PTCH

Ethylenedicysteine (EC) was selected as a chelator for PTCH conjugation. Sodium bicarbonate (1 N, 1 mL) was added to a stirred solution of EC (5 mg, 0.019 mmol). To this colorless solution, sulfo-NHS (4 mg, 0.019 mmol) and EC (5 mg, 0.019 mmol) were added. PTCH (0.3 mg) was then added. The mixture was stirred at room temperature for 24 hours. The mixture was dialyzed for 48 hours with a cutoff at molecular weight 10,000 Da. After dialysis, the product was freeze-dried, with the product in the salt form weighing 0.5 mg.

^99m^Tc-pertechnetate was obtained from Mallinckrodt (Houston, TX). Radiosynthesis of ^99m^Tc-EC-PTCH was achieved by adding the required amount of ^99m^Tc-pertechnetate into EC-PTCH (0.1 mg) and tin chloride (II) (SnCl_2_, 100 mg). The mixture was loaded on a sephadex gel column (PD-10, G-25) (Sigma Chemical Company, St. Louis, MO) and eluted with phosphate-buffered saline (pH 7.4). One milliliter of each fraction was collected. The product was collected at fraction 3, with a 70% yield. Radiochemical purity was assessed by Radio-TLC (BioScan, Washington, DC) using saline as an eluant.

### 2.3. Cell Lines and Culture Conditions

The human cell lines T47-D, SKBR3, and MCF-7 were obtained from the American Type Tissue Company (ATCC) and cultured in DMEM with 10% fetal bovine serum (Atlanta Biologicals, Flowery Branch, GA) and 1% antibiotic-antimycotic (Invitrogen Life Technologies, Grand Island, NY Life Technologies, Grand Island, NY). The human cell line SUM159 was obtained from Asterand (Detroit, MI) and cultured in DMEM containing 1 *μ*g/mL hydrocortisone (Invitrogen Life Technologies, Grand Island, NY Life Technologies, Grand Island, NY), 5 *μ*g/mL insulin (Invitrogen Life Technologies, Grand Island, NY), and 1% antibiotic/antimycotic. The rat breast cancer cell line 13762 was derived from a tumor induced in a Fischer-344 rat by giving an oral dose of 7,12-dimethylbenz[a]anthracene [3], and the cells were cultured in RPMI-1640 medium, supplemented with 10% (vol : vol) fetal bovine serum and 1% antibiotic-antimycotic. For mammosphere assays, cells were cultured in MEM media supplemented with 1X B27 (Invitrogen Life Technologies, Grand Island, NY), 20 ng/mL epidermal growth factor (EGF; Invitrogen Life Technologies, Grand Island, NY), and 20 ng/mL basic fibroblast growth factor (bFGF; Invitrogen Life Technologies, Grand Island, NY) and seeded into ultralow attachment plates (Corning Life Sciences, Salt Lake City, UT). Cells were grown for 7–10 days and spheres were obtained. All cells were cultured at 37°C in a humidified atmosphere containing 5% carbon dioxide.

### 2.4. Survival Assays

Breast cancer cell lines were seeded into 96-well plates at a density of 5,000–7,000 cells per well. Cells were grown overnight and media were replaced with culture media containing unlabeled peptides A and B at the indicated concentrations. Cells were cultured for an additional 48 hours and survival was determined using the MTT-based Cell Proliferation Assay (Biotium). Data is expressed as %treated/untreated.

### 2.5. Fluorescence Microscopy

Breast cancer cell lines were seeded onto chamber slides (Nunc, Roskilde, Denmark) and grown overnight. For sphere assays, cells were seeded in 3D media as described above in 96-well low attachment plates at a density of 100–1000 cells per well and cultured for 7–10 days until spheres were formed. Spheres were filtered using a cell strainer and replated into low attachment plates. Cells or spheres were treated with 100 nM peptide A or B and incubated for 2 hours. Media were removed and cells or spheres were washed two times with 1X PBS. Following washes, 1 mL of 1X PBS was added to the slide or plate and cells or spheres were analyzed by fluorescence microscopy using a Zeiss motorized AxioObserver Z1 microscope. For co-localization experiments, cells were seeded at a density of 7000 cells per well in a chamber slide and cultured overnight. Cells were incubated with 100nM peptide and incubated for 2 hours. Cells were fixed in methanol at -20 degrees Celsius for 5 min, and blocked with PBS containing 10% goat serum. Cells were stained with anti-PTCH antibody (Santa Cruz Biotechnology, Dallas, TX) overnight at 4 degrees Celsius, washed 3 time in 1XPBS and incubated with an anti-rabbit Alexa-555 secondary antibody for 1 hour at RT. Slides were washed 3 times with PBS and stained with DAPI Prolong Gold (Invitrogen Life Technologies, Grand Island, NY). Slides were analyzed using a Zeiss motorized AxioObserver Z1 microscope.

### 2.6. Uptake Studies

To measure uptake of the FITC-tagged peptide, cells were plated at a density of 5,000–10,000 cells per well in a 24-well plate and grown overnight. For sphere assays, cells were plated as described above. Cells or spheres were treated with 100 nM peptide and incubated for 2 hours. Cells or spheres were washed 3X with PBS, trypsinized, and resuspended in 500 *μ*L culture media. Cells were counted using a Countess automated cell counter (Invitrogen Life Technologies, Grand Island, NY Life Technologies, Grand Island, NY) after staining with trypan blue (Invitrogen Life Technologies, Grand Island, NY Life Technologies, Grand Island, NY). Next, 100 *μ*L of cell suspension was transferred to black polystyrene 96-well plates. Fluorescence was measured at 485 nm excitation and 535 nm emission. Uptake is reported as mean fluorescence intensity per 1,000 cells.

The rat breast carcinoma cell line 13762 and the human breast cancer cell lines SUM159 and MDA-IBC3 were used for the* in vitro* radiotracer uptake. One day before the uptake experiment, 2 × 10^5^ cells/well of each cell line were seeded in six-well plates and incubated at 37°C in 5% CO_2_ under humidified conditions. The following day, 300 kBq of ^99m^Tc-EC-peptide A or ^99m^Tc-EC was added with 2 mL of the appropriate media to each of the wells. The cells were incubated for 30 minutes, 1 hour, 2 hours, or 4 hours, after which the media was aspirated, cells were washed twice with PBS, and then cells were suspended with trypsin. Radioactivity of collected cells was measured on a gamma counter (Packard) with an energy window of 126–154 keV for ^99m^Tc, and percent uptake was calculated by using an appropriate standard. Percent uptake was then normalized to milligrams of protein in the sample, where the protein concentration was measured using the Bradford method (Bio-Rad Laboratories, Berkeley, CA). Each sample was run in triplicate, with error bars indicating standard deviation.

### 2.7. Animal Model and Chemotherapy Treatment

All animal work was carried out in the Small Animal Imaging Facility (SAIF) at the University of Texas M.D. Anderson Cancer Center under an approved Institutional Animal Care and Use Committee (IACUC) protocol. Female Fischer 344 rats (150 ± 25 g, *n* = 6) (Harlan Sprague-Dawley, Indianapolis, IN) were inoculated subcutaneously with 0.1 mL of a 13762 breast carcinoma cell suspension (10^5^ cells/rat of a breast tumor cell line specific to Fischer rats) into the hind legs using 25-gauge needles. Studies were performed 12–14 days after inoculation when tumors reached approximately 1 cm in diameter. For treatment studies, rats were injected with 20 mg/kg paclitaxel and reimaged 7 days later. After the posttreatment scan, tumors were removed and formalin sections were made. Control tumors were taken from untreated mice 13 days after inoculation. Sections were stained with an anti-patched antibody (Santa Cruz) using a peroxide-based immunohistochemical detection kit (Dako) according to the manufacturer's instructions.

### 2.8. Planar Imaging

Planar scintigraphic images were obtained using M-CAM (Siemens Medical Solutions, Hoffman Estates, IL) equipped with a low energy high resolution (LEHR) collimator. Anesthetized breast tumor-bearing rats were injected intravenously with ^99m^Tc-EC-peptide A (0.3 mg/rat, 300 *μ*Ci/rat; *n* = 3) before and 7 days after paclitaxel treatment. ^99m^Tc-EC (0.15 mg/rat, 300 *μ*Ci/rat; *n* = 3) was used as a control. The images were acquired at 1 hr, 2 hr, and 4 hr after administration of radiotracers. Computer outlined regions of interest (ROIs in counts per pixel) between tumor and muscle were used to calculate tumor-to-muscle (T/M) ratios.

### 2.9. Statistical Analysis

Statistical analysis was performed using Graph Pad Prism 6 software (Graph Pad, La Jolla, CA) using ANOVA or unpaired *t*-test. For all tests, *P* values less than 0.05 were considered to be significant.

## 3. Results

### 3.1. Growth Inhibitory Effect of Peptides A and B in Breast Cancer Lines

**Figure 1:**
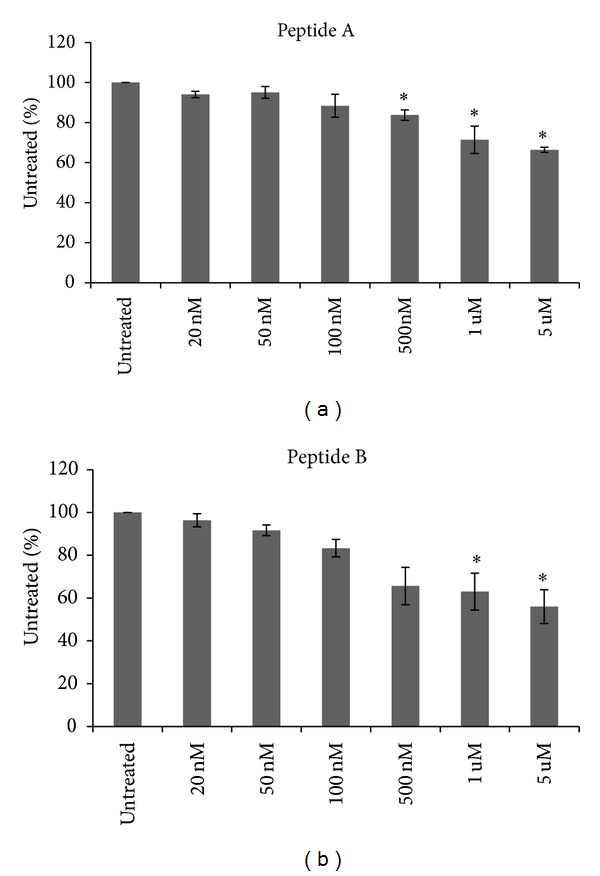
Patched-binding peptides decrease growth of the SKBR3 breast cancer cell line. Using the MTT assay, peptides A and B administered to SKBR3 significantly decreased growth compared to untreated cells. Error bars represent standard deviation. Significance is represented by asterisk. *P ≤ 0.05.

Inhibition of hedgehog signaling has been shown to decrease growth and survival of breast cancer cells [2, 4]. Antibodies that disrupt the binding of sonic hedgehog to the PTCH receptor have also been reported to inhibit breast cancer growth [5]. The PTCH-binding peptides, referred to, in this paper, as peptides A and B, have previously been shown to decrease hedgehog-dependent growth of pancreatic cancer cell lines. Therefore, we sought to determine their effect on breast cancer cell lines. As shown in Figure 1, treatment of SkBr3 breast cancer cell lines with peptides A and B resulted in significant growth inhibition at higher concentrations. Minimal effect was observed at lower concentrations.

### 3.2. Peptide Uptake in Breast Cancer Cell Lines and Mammospheres

**Figure 2:**
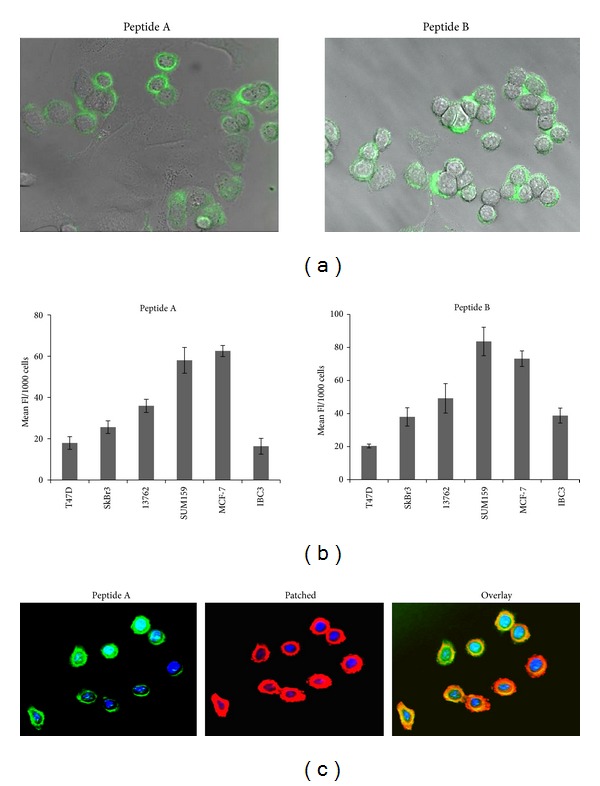
Patched-binding peptides have significant uptake in breast cancer cell lines. (a) Fluorescence microscopy of FITC-labeled peptides A and B in SKBR3 breast cancer cells. (b) Quantification of FITC-peptides A and B uptake in breast cancer cell lines. (c) Fluorescence microscopy of SKBR3 cells showing colocalization of peptide B (green) with the PTCH receptor (red). Colocalization appears as yellow staining in the image overlay.

To validate the PTCH-binding peptides A and B as ligands to detect breast cancer cells, we evaluated the cellular uptake of the peptides labeled with FITC. Fluorescence microscopy of breast cancer cell lines revealed uptake of the FITC-tagged peptides. As shown in Figure 2(a), cytosolic fluorescence was observed 24 hours after treatment of the breast cancer cell line SKBR3 with peptide A or peptide B. To quantify these findings, uptake studies were performed in a panel of breast cancer cell lines and fluorescence intensity was measured. As shown in Figure 2(b), significant uptake of the fluorescent peptides was observed. Furthermore, fluorescent intensity corresponded to PTCH expression as previously reported [1], suggesting that binding is specific to the PTCH receptor. To further confirm colocalization of PTCH-binding peptides with PTCH receptor expression, we performed fluorescence microscopy using anti-PTCH antibodies on cells treated with FITC-labeled PTCH-binding peptides. As shown in Figure 2(c), uptake of PTCH-binding peptides (green) colocalized with PTCH receptor expression (red).

**Figure 3:**
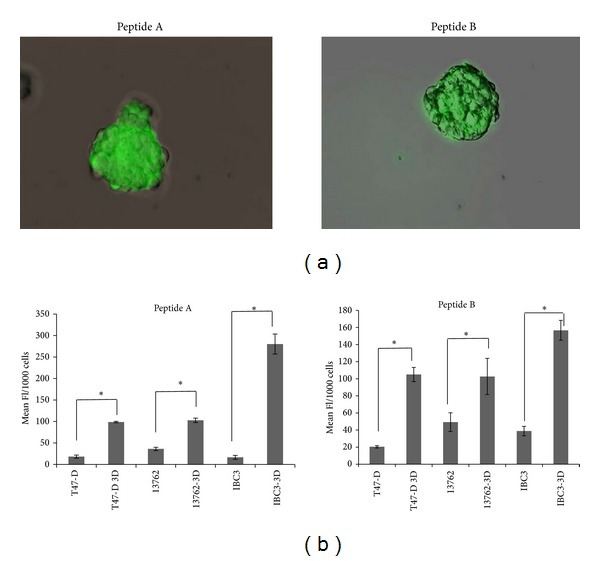
Uptake of patched-binding peptides is increased in mammospheres from breast cancer cell lines. (a) Fluorescence microscopy of FITC-labeled peptides A and B in mammospheres from T47-D. (b) Quantification of FITC-peptides A and B uptake in breast cancer cell lines in monolayer and mammosphere cultures. Uptake was significantly higher in cells cultured in mammosphere promoting conditions (3 dimensional cultures) than in those grown in standard monolayer conditions. Significance is represented by asterisk. *P ≤ 0.01.

Hedgehog pathway members PTCH, Gli-1, and Gli-2 have been reported to be more highly expressed in normal mammary stem cells and their malignant counterparts, breast cancer stem cells, compared to more differentiated breast cancer cells [6]. High expression of the PTCH receptor has been reported in breast cancer cells cultured in stem cell-enriching conditions (mammospheres, 3 dimensional cultures). Consistent with these findings, an increase in peptide uptake was observed in mammospheres compared to 2 dimensional monolayer cultures (Figure 3). These data indicate that PTCH-binding peptides may provide a method of targeting breast cancer stem cells.

### 3.3. Synthesis and Radiolabeling of EC-Peptide A

**Figure 4:**
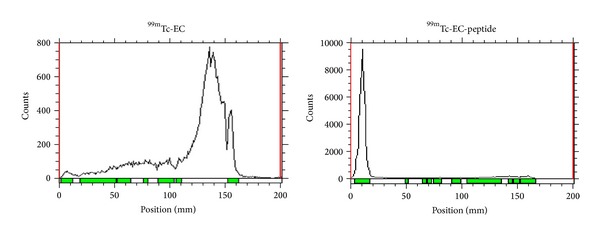
Radiochemical purity of ^99m^Tc-EC and ^99m^Tc-EC-peptide A. Radiochemical purity was determined by RadioTLC with saline as the mobile phase.

To establish the uptake of PTCH-binding peptides* in vivo*, we synthesized chelator-peptide conjugates that could be radiolabeled with ^99m^Tc for gamma scintigraphy. A simple and efficient synthesis of ^99m^Tc EC-PTCH was developed. EC was conjugated to the lysine residue of peptide A. ^99m^Tc EC-PTCH was found to be radiochemically pure (100%, Figure 4).

### 3.4. *In Vitro* Uptake Studies

**Figure 5:**
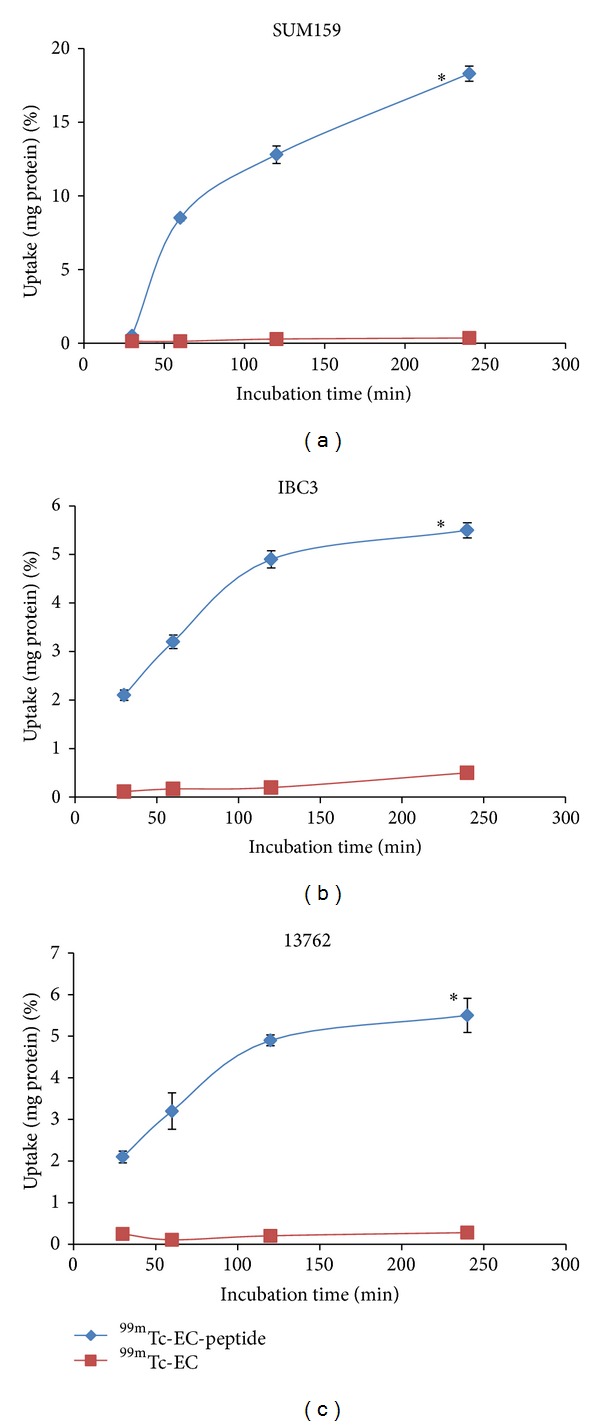
*In vitro* uptake of ^99m^Tc-EC-peptide A. Results of* in vitro* assays showing significant uptake of peptide A compared to ^99m^Tc-EC control in (a) SUM159, (b) MDA-IBC3, and (c) 13762 breast cancer cell lines. Data is represented as % uptake per mg/protein. Error bars represent standard deviations. Significance is represented by asterisk. *P ≤ 0.001.

*In vitro* cellular uptake of the ^99m^Tc-conjugated peptide was performed in three breast cancer cell lines: SUM159, MDA-IBC3, and 13762. As shown in Figure 5, cellular uptake of the peptide conjugate was significantly higher than that of the chelator alone in all lines. Similar to the data for the FITC-tagged peptide, the SUM159 line showed the highest radiotracer uptake with approximately 18% uptake per mg protein at 4 hours (Figure 5(a)). Significant uptake of the radiotracer was also observed in two other lines, increasing steadily in MDA-IBC3 (Figure 5(b)) and reaching saturation after 1-2 hours in 13762 (Figure 5(c)).

### 3.5. *In Vivo* Imaging

**Figure 6:**
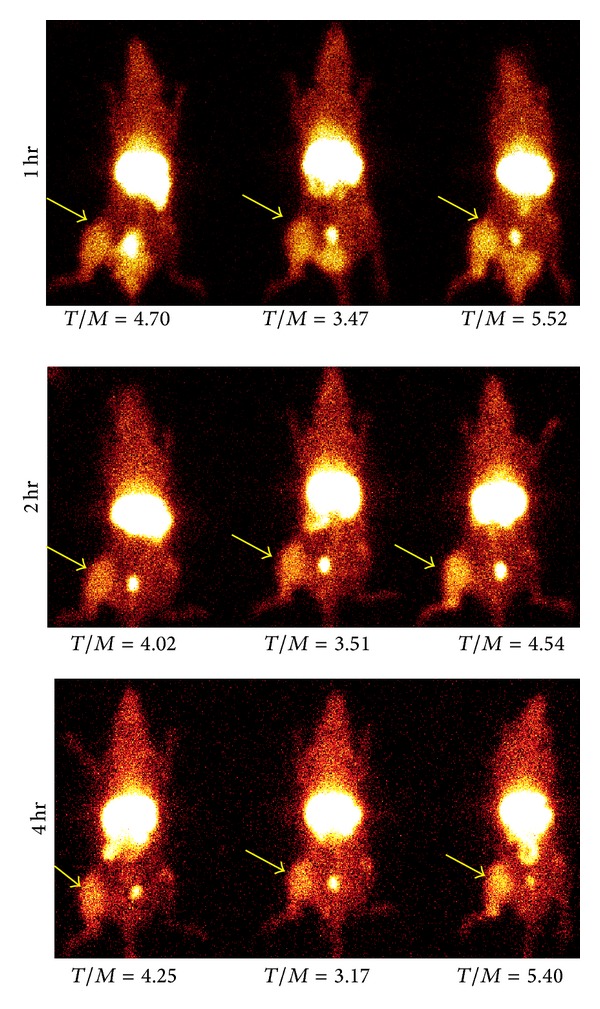
*In vivo* uptake of ^99m^Tc-EC-peptide A in rats bearing 13762 breast carcinoma xenografts. Tumor-to-muscle (T/M) ratios are given for 3 separate rats at multiple timepoints after injection of the radiotracer. Arrows indicate tumor location.

To investigate the utility of peptide imaging of the PTCH receptor in breast cancer, planar scintigraphy was performed in a rat model of breast cancer using ^99m^Tc-EC-peptide A. Fisher rats were inoculated with the mammary carcinoma cell line 13762, and after tumors grew for two weeks, rats were injected with approximately 300 *μ*Ci of the ^99m^Tc-labeled peptide. Planar scintigraphy was conducted at 1, 2, and 4 hours after injection of the radiolabeled peptide, and tumor-to-muscle ratios were calculated. An average tumor-to-muscle ratio of 4.5 ± 0.07 was obtained at 1 hour. Significant retention of the peptide was observed in the tumor tissue up to 4 hours after injection (Figure 6).

**Figure 7:**
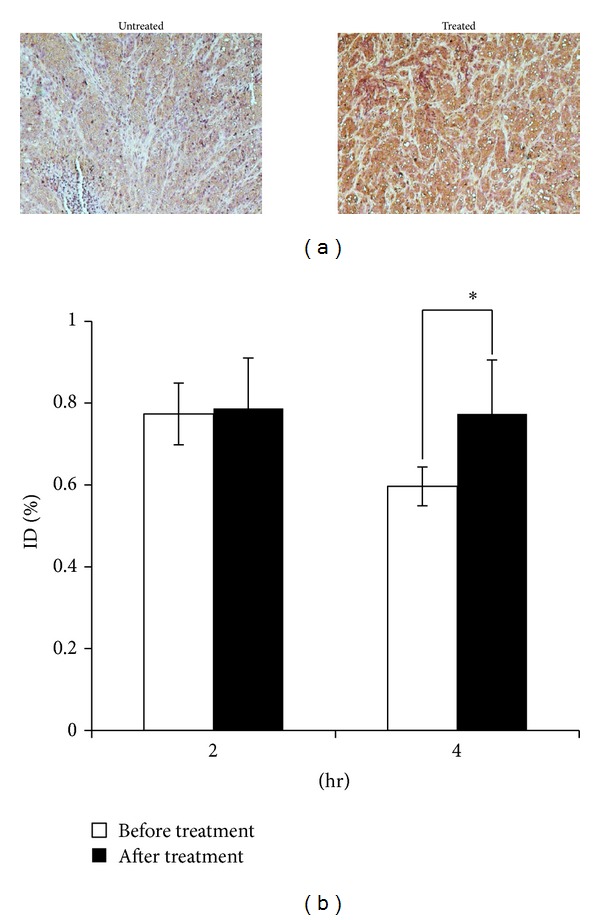
PTCH expression is increased in breast cancer xenografts after chemotherapy treatment. (a) Immunohistochemical detection of PTCH before and after treatment with paclitaxel. (b) Tumor uptake of ^99m^Tc-EC-peptide A is increased after treatment with chemotherapy. Data represents % injected dose in the ROI of the tumor. Error bars represent standard deviation. Significance is represented by asterisk. *P ≤ 0.05.

Several studies have reported that hedgehog signaling induces resistance to chemotherapy [4, 7, 8]. Therefore, we expect that residual cells which remain after treatment with chemotherapy would have high expression of hedgehog pathway members. We examined PTCH expression in tumor xenografts before and after treatment with paclitaxel. As shown in Figure 7(a), an increase in PTCH protein expression is observed in the residual tumor seven days after treatment. Although there was a decrease in tumor volume after treatment, planar imaging with ^99m^Tc-EC-peptide A revealed no significant decrease in tumor accumulation of the peptide (Figure 7(b)). These findings suggest that PTCH receptor imaging may provide a useful method to assess resistant tumor tissue after chemotherapy treatment.

## 4. Discussion

Neoadjuvant chemotherapy is commonly prescribed for treatment of invasive or large tumors to allow for breast-conserving surgery. However, there is currently no reliable method to noninvasively follow response to chemotherapy. It is unclear whether the current standard for clinical imaging, ^18^F-FDG PET, is predictive of treatment response due to false positive results following treatment.* In vitro* and* in vivo* studies have demonstrated high FDG uptake in inflammatory lesions [5]. Increased FDG uptake in macrophages and neutrophils caused by treatment-induced inflammation has also been reported [9, 10].

We show that* in vivo* imaging with ^99m^Tc-PTCH peptides may offer an alternative method to follow treatment response and allow for tumor-specific imaging prior to and immediately after chemotherapy treatment. Our data suggest that peptides which bind to the ligand docking site of the hedgehog receptor, PTCH, are localized to breast cancers* in vivo*. Furthermore, we show that PTCH receptor expression is increased after paclitaxel treatment in a rat model of breast cancer. These results indicate that PTCH-positive, treatment-resistant cells may be enriched after chemotherapy. In addition to tumor uptake, significant uptake of the peptide was observed in liver and kidney tissues. This may be due to clearance of the peptide and the FITC tag. Additionally, liver uptake may be due to low level endogenous expression of the PTCH receptor by liver tissue. Although our work and that of others [2] suggest that PTCH docking peptides specifically target the PTCH receptor on cancer cells, binding to other cell surface receptors cannot be ruled out.

The cancer stem cell hypothesis states that tumors consist of a heterogeneous population of cells, including both rapidly dividing, differentiated cells that can be effectively targeted by chemotherapy and relatively resistant stem-like cells [11]. Previous studies have reported that breast cancer stem cells express high levels of the PTCH receptor and that hedgehog signaling is required for the growth of these cells [6]. Our data indicate that PTCH-binding peptides have higher uptake in cells cultured under stem cell-promoting conditions (mammospheres) and may serve as a ligand to detect and target this cell population. Additionally, our findings suggest that PTCH receptor-positive cells are resistant to chemotherapy and that ^99m^Tc-peptide A may be a useful agent for the detection of treatment-resistant breast cancer cells with active hedgehog signaling.

PTCH-blocking peptides have been shown to decrease growth of pancreatic cancer cell lines [2]. Similar to previous studies, we show that treatment of breast cancer cell lines with PTCH-binding peptides decreases growth of breast cancer cell lines. While our preliminary results suggest that PTCH-binding peptides may slow growth of breast cancer, further study is needed to validate the therapeutic effect. Our data also indicate that further evaluation of the effect of PTCH-binding peptides on tumor detection, growth, and survival in orthotopic models of breast cancer is warranted. These peptides may serve as useful theranostics which may be used to both image and treat breast cancer.
